# CD8+ T Cells and PD-L1 Expression as Prognostic Indicators in a Low Prevalence of HPV-Associated Oropharyngeal Squamous Cell Carcinoma

**DOI:** 10.3390/curroncol30020111

**Published:** 2023-01-20

**Authors:** Kawita Atipas, Natthawadee Laokulrath, Janjira Petsuksiri, Narin Ratanaprasert, Warut Pongsapich

**Affiliations:** 1 Department of Otorhinolaryngology, Faculty of Medicine Siriraj Hospital, Mahidol University, Bangkok 10700, Thailand; 2 Department of Pathology, Faculty of Medicine Siriraj Hospital, Mahidol University, Bangkok 10700, Thailand; 3 Division of Radiation Oncology, Department of Radiology, Faculty of Medicine Siriraj Hospital, Mahidol University, Bangkok 10700, Thailand

**Keywords:** oropharyngeal squamous cell carcinoma (OPSCC), survival, human papillomavirus (HPV), p16, CD8, PD-L1

## Abstract

Human papillomavirus (HPV) infection detected in oropharyngeal squamous cell carcinoma (OPSCC) is associated with a better survival outcome from previous literature. However, Thailand and several Asian countries have a low prevalence of HPV-associated OPSCC and, therefore, have a low positive rate of immunostaining with p16. Tumor microenvironments (TME), including tumor-infiltrating CD8+ lymphocytes (CD8+ TIL) and programmed death ligand 1 (PD-L1), are proposed as significant prognostic indicators in addition to p16. Objectives: Explore the expression p16, CD8+ TIL, and PD-L1 and its value as prognostic indicators for overall survival (OS) in patients with OPSCC. Materials and Methods: Data from patients with OPSCC diagnosed from 2012 to 2018 were recovered from medical records and national registry. All available glass slides and slides of immunohistochemistry (IHC) of p16, CD8, and PD-L1 were reviewed. The TME was classified into four types according to the expression pattern of PD-L1 and CD8+TIL. Overall survival (OS) was assessed using the Kaplan–Meier method and Cox regression model analysis. Results: In 160 OPSCC patients, p16 was positive in 27 (16.88%). The density of CD8+ TIL was higher in the p16+ and PD-L1+ groups (*p* = 0.005, 0.039); however, there was no association between p16 and the status of PD-L1. P16 and CD8+ TIL were significant prognostic factors for better OS (*p* = 0.007, 0.001), but not PD-L1 status (*p* = 0.317). Among the four types of TME, carcinoma showed mainly type IV TME (PD-L1−/TIL+), while OPSCCs with type I TME (PD-L1+/TIL+) had the best survival outcome. Conclusions: The positivity of p16 and the density of CD8+ TIL were associated with better OS in OPSCC, while the status of PD-L1 was not significantly related to OS. OPSCC with type I TME (PD-L1+/TIL+) showed the best prognosis of all types of TME.

## 1. Introduction

Oropharyngeal squamous cell carcinoma (OPSCC) has been in the spotlight with a rapidly increasing incidence over the past decade. Evidence shows that high-risk human papillomavirus (HPV) infection is a causative pathogen of OPSCC and has a favorable prognostic value for patients with OPSCC [1]. The attributable fraction for HPV is greater than 60% in the United States and 40% in Europe [2,3]. However, in Thailand, the reported prevalence of high-risk HPV infection in OPSCC is only about 8.7–15.6% [4,5,6,7].

Although HPV-driven tumorigenesis is associated with the chronic inflammatory process of viral infection; the host immune responses are possibly different from smoking carcinogenesis. The immune system plays a vital role in tumor eradication, so cancer cells develop mechanisms to avoid detection or dysregulate the immune system. These mechanisms include the development of tolerance to T cells, the alteration of HLA class I, the inhibition of inflammatory cytokines, and the evasion of the immune checkpoint [8]. Thus, components of the tumor microenvironment (TME), such as lymphocytes, macrophages, or immune checkpoints, are believed to play an essential role in the inhibition and/or development of cancer cells [9,10].

CD8+ lymphocytes are cytotoxic T-lymphocytes that function as an antigen-specific immune response. These T-lymphocytes play a role in the tumor microenvironment by increasing antitumor immune responses. In HPV-associated cancers, many studies have observed a high density of tumor-infiltrating CD8+ lymphocytes (CD8+ TIL), and an increase in CD8+ TIL leads to better OS (overall survival) outcomes [11,12,13].

Programmed death protein 1 (PD-1) is a transmembrane receptor expressed by T cells, B cells, monocytes, and dendritic cells, which plays a role in the immune checkpoint cascade. Binding of PD-1 to its ligands, the programmed death ligand 1 (PD-L1) on tumor cells, helps them escape immune surveillance [8]. Tumor PD-L1 is associated with various prognoses [14,15,16]. The expression of PD-L1 on both cancer cells and immune cells is shown to be associated with survival outcomes and responses to immune checkpoint inhibitors [16,17]. In particular, the expression is controlled by many factors related to oncogenic pathways or inflammatory pathways such as IFN- γ [18,19]. The up-regulation of PD-L1 on tumor cells in the oropharynx is an adaptive immune response during chronic viral infection [15,20]. In some studies, high expression of PD-L1 is observed in HPV-associated cancers [16], with malignant transformation in deep tonsillar crypts where HPV infection often occurs [20]. 

The low prevalence of HPV-associated OPSCC results in a low positive rate of p16 expression and a higher discordant rate between p16 and PCR for HPV DNA when compared to western countries [7], so the role of p16 as the sole prognostic factor may be less significant compared to areas with a high prevalence of HPV. Ang et al. originally suggested a risk classification based on HPV and smoking statuses [21]. A low prevalence of HPV and a high rate of smoking in the Thai population may result in fewer patients in the low-risk group, so the classification using only these two factors may not fully reflect the risk of death in this setting. This study aims to demonstrate the role of CD8+TIL and PD-L1 as additional prognostic factors for survival outcomes in a setting with a low prevalence of HPV. 

## 2. Materials and Methods

### 2.1. Study Population and Design

Patients with OPSCC aged 18 years or older treated at Siriraj Hospital between January 2012 and December 2018 were included. Patients with all stages of the disease were included, except for recurrent cases, regardless of the treatment received. Most of the patients received definitive chemoradiation, followed by radiation alone and surgery with adjuvant chemoradiation. Patient data was collected from electronic medical records. The study endpoint was the OS outcome, defined as the time from diagnosis to death from any cause or the end of the study period on 30 April 2020. The date of death was acquired from the Thai national registry. All available glass slides and the additional IHC staining of p16, CD8, and PD-L1 performed in formalin-fixed paraffin-embedded tumor blocks were reviewed and evaluated by a pathologist. This study was approved by the Siriraj Institutional Review Board, (EC2) 336/2562.

### 2.2. Immunohistochemical Method

**Figure 1:**
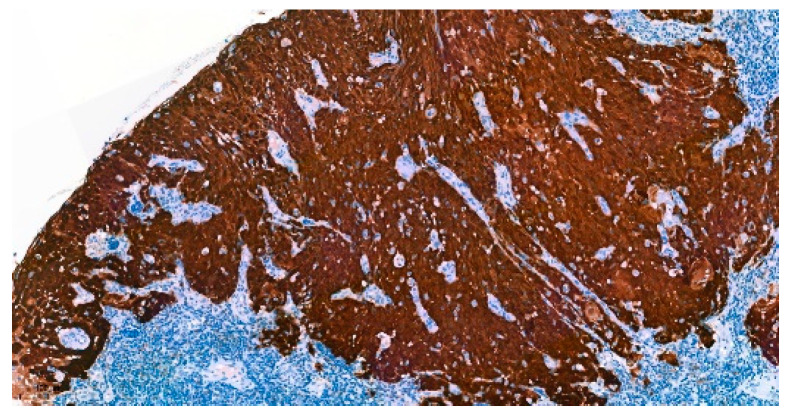
Immunohistochemistry of p16-positive OPSCC (100×).

Formalin-fixed paraffin-embedded blocks of tumor specimens were analyzed for IHC of p16, CD8, and PD-L1. Paraffin-embedded tumor sections were deparaffinized and incubated with monoclonal antibodies. IHC of p16 was performed using a mouse monoclonal primary antibody against p16INK4a (CINtec^®^ Histology, Ventana, AZ, USA) (Figure 1). The positive expression of p16 was interpreted with strong and diffuse nuclear and cytoplasmic staining (block staining) ≥ 70% of tumor cells [22]. 

**Figure 2:**
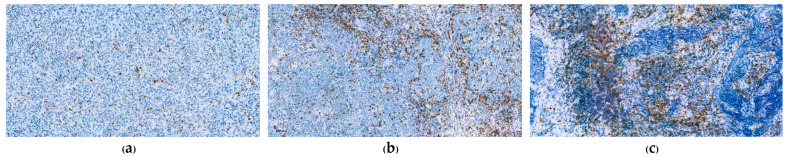
Immunohistochemical staining for CD8 (100×). (**a**) CD8 ≥ 1% but < 5%; (**b**) CD8 ≥ 5% but <10%; (**c**) CD8 ≥ 10%.

CD8 IHC was performed using CD8 (C8/144B, Cell Marque) mouse monoclonal primary antibody. Expression of CD8 was calculated as the percentage of CD8+ lymphocytes infiltrating the tumor divided by the total number of tumor cells and classified into four groups (Figure 2): CD8 ≥ 10%, CD8 ≥ 5% but < 10%, CD8 ≥ 1% but < 5%, and CD8 < 1% [11].

**Figure 3:**
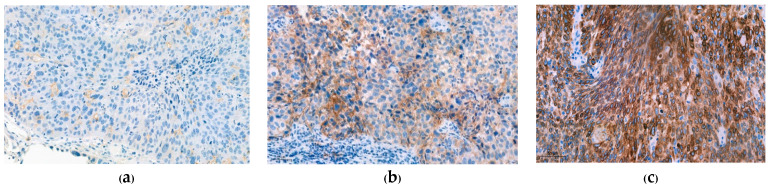
Immunohistochemical staining for PD-L1 (200×). (**a**) CPS ≥ 1 but < 20 with weak intensity (1+); (**b**) CPS ≥ 20 with moderate intensity (2+); (**c**) CPS ≥ 20 with strong intensity (3+).

PD-L1 expression in a tissue microarray by IHC used a mouse IgG antibody against PD-L1 (22C3, PD-L1 IHC 22C3 pharmDx). The expression of PD-L1 was evaluated using the Combined Positive Score (CPS) and classified into three groups: CPS < 1, CPS ≥ 1 but < 20, and CPS ≥ 20, according to the interpretation manual (Figure 3). Staining intensity was also visually assessed and manually scored as 1+ (weak intensity), 2+ (moderate intensity), and 3+ (strong intensity).

### 2.3. Tumor Microenvironment

Tumor microenvironments were classified into 4 types as suggested by Teng et al. by the status of PD-L1 and CD8+TIL which was type I (PD-L1+/TIL+, adaptive immune resistance), type II (PD-L1−/TIL−, immunological ignorance), type III (PD-L1+/TIL−, intrinsic induction), and type IV (PD-L1−/TIL+, tolerance) [23]. PD-L1+ was defined as CPS ≥ 1 and TIL+ was defined as CD8 ≥ 1% in this study.

### 2.4. Statistical Analysis

Baseline characteristics were presented as mean ± standard deviation (SD) for continuous data and number (percentage) for categorical data. Continuous data was compared using *t* tests. Chi-square and Fisher’s exact tests were applied to the categorical data. OS rates were assessed using the Kaplan–Meier method, with a comparison between the two groups using the log-rank test. Cox proportional-hazards models were used to estimate the hazard ratio (HR). Statistical analysis was performed using the Statistical Package for the Social Sciences (SPSS) version 22.0 software. A *p*-value < 0.05 was considered statistically significant.

## 3. Results

### 3.1. Immunohistochemical Analysis

160 patients met the inclusion criteria and were enrolled in this study. Of these, immunohistochemical staining of p16 was positive in 27 patients (16.88%). The results of CD8+TIL density and the Combined Positive Score (CPS) of PD-L1 expression are shown in Table 1. The usual cut-off point for pembrolizumab treatment was CPS ≥ 1, which represented 29.1% of the study groups. However, none of the patients in this study were treated with immune checkpoint inhibitors. PD-L1 intensity was assessed in all cases with CPS ≥ 1.

The high density of CD8+ TIL was associated with positivity of p16 expression (*p* = 0.005) and PD-L1 expression (*p* = 0.039). However, the expression of PD-L1 was not statistically associated with the status of p16 (*p* = 0.596), as shown in Table 2.

### 3.2. Patient Characteristics

Most of the patients (93.75%) were men and 91.08% were smokers. Of these, 7.01% had no history of smoking or alcohol consumption. Furthermore, patients with p16+ OPSCC were younger than those with p16− as expected (*p* = 0.045). High CD8+ TIL and positive PD-L1 were associated with a lower rate of metastasis (*p* = 0.040, 0.035). The baseline characteristics according to each IHC are shown in Table 3. 

### 3.3. Survival Outcomes

The median follow-up time was 1.38 years (range, 0.06–8.26), 1.34 years in p16− OPSCC, and 2.43 years in p16+ OPSCC. Kaplan–Meier analysis yielded a three-year OS of 28.9% and 56.7% for p16− and p16+ patients, respectively. The difference in OS between both groups was statistically significant (*p* = 0.007). 

**Figure 4:**
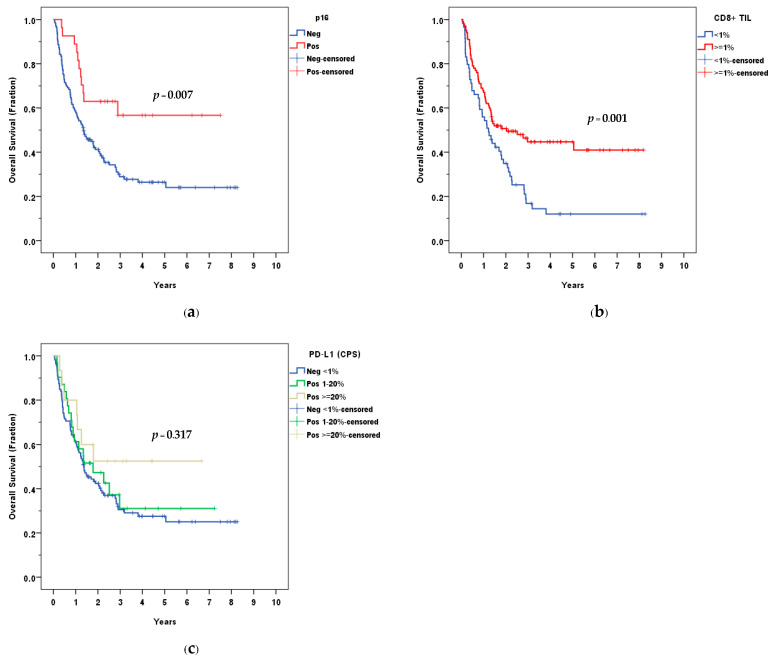
Overall survival (OS) according to: (**a**) p16; (**b**) CD8+ TIL; (**c**) PD-L1 (CPS).

The median survival time was 1.41 years (95% CI, 0.88–1.95). The median survival time was 1.35 years (95% CI, 0.87–1.83) in the p16−group, while no median survival was reached in the p16+ group. The Kaplan–Meier survival curve is shown in Figure 4a.

OS was not significantly different according to the level of expression of PD-L1 (*p* = 0.317) (Figure 4c); while the high density of CD8+ TIL was associated with better OS, using a percentage of CD8+ TIL at ≥1%, ≥ 5%, and ≥10% as cut-off points (*p* = 0.001, 0.009, 0.006). The OS between CD8+ TIL < 1% and ≥1% was demonstrated in Figure 4b. When analyzing only the p16– group, a high density of CD8+ TIL was associated with better OS using ≥1% as the cut-off point (*p* = 0.015). 

**Figure A1:**
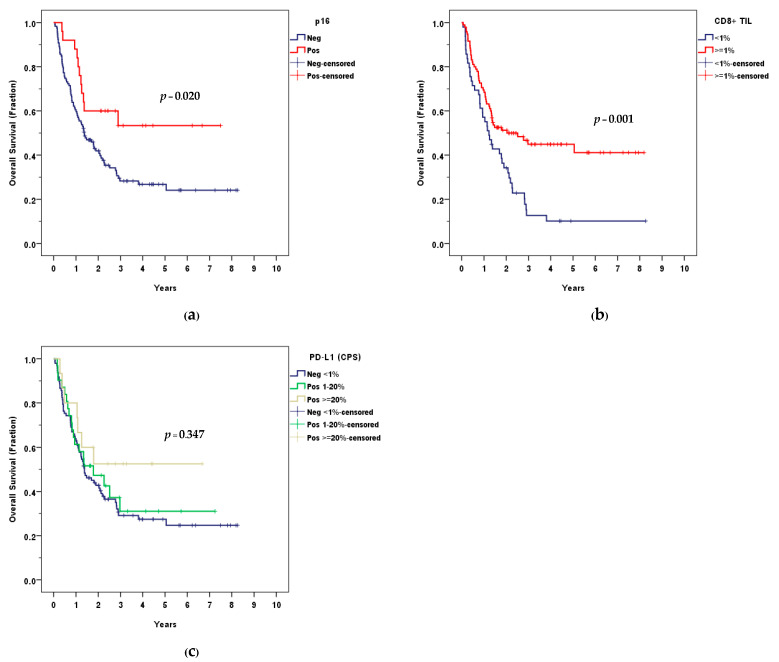
Overall survival (OS) of a subgroup analysis without patients in stage I and patients with distant metastases according to: (**a**) p16; (**b**) CD8+ TIL; (**c**) PD-L1 (CPS).

A subgroup analysis excluding patients in stage I (AJCC 7th) and patients with distant metastases was performed. Similar to the findings from the overall study population, the positivity of p16 and the high density of CD8+ TIL were associated with better OS (*p* = 0.020, 0.001). Again, OS was not significantly different according to the level of expression of PD-L1 (*p* = 0.347). The Kaplan–Meier survival curve is shown in Appendix A (Figure A1). 

High stages, negative p16, and low CD8+ TIL density were predictors of poor OS outcomes in multivariate analysis (HR 2.43, 95% CI 1.11–5.32, *p* = 0.026; HR 1.98, 95% CI 1.05–3.71, *p* = 0.034; HR 1.77, 95% CI 1.18–2.67, *p* = 0.006) as shown in Table 4.

### 3.4. Tumor Microenvironment

**Figure 5:**
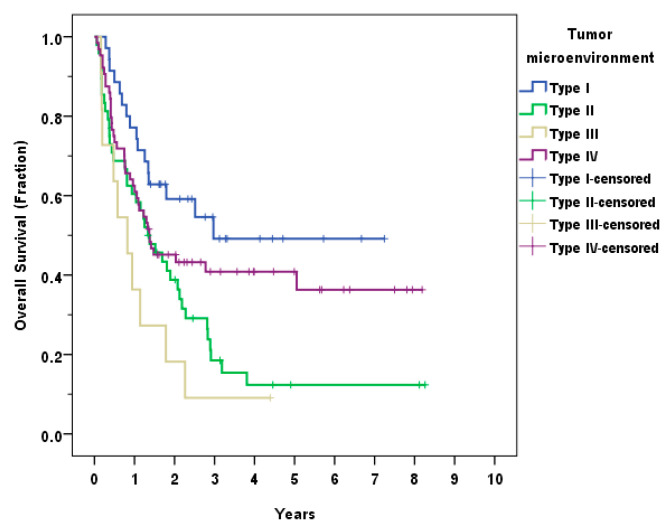
Overall survival (OS) according to the type of tumor microenvironment (TME).

The patients were classified into four groups according to the TME as follows: type I (PD-L1+/TIL+, adaptive immune resistance) 35 (22.2%), type II (PD-L1−/TIL−, immunological ignorance) 48 (30.4%), type III (PD-L1+/TIL−, intrinsic induction) 11 (7.0%), and type IV (PD-L1−/TIL+, tolerance) 64 (40.5%). The OS differed significantly between the four types (*p* = 0.004). The group with the best survival outcome was type I, followed by type IV, II and III, respectively. The Kaplan–Meier analysis of the TME types is shown in Figure 5.

## 4. Discussion

The role of TME has been widely studied in recent years, especially for OPSCC, where HPV is known to be one of the causal factors. HPV-associated OPSCC demonstrated favorable survival and treatment outcomes in the previous literature [21,24,25]. The favorable survival outcome was also observed in recurrent or metastatic head and neck squamous cell carcinoma (HNSCC) [26]. The higher density of TILs was observed in HPV+ OPSCC; the TIL is a part of adaptive immunity and has a protective effect against tumor activity, as evidenced by a better outcome in the high TIL subgroup of patients with OPSCC [27,28]. However, similar to reports from many Asian countries, the prevalence of HPV+ OPSCC in Thailand was considerably lower compared to those in the United States and European countries. In our study, the rate of p16+ from tumor specimens was 16.88%, while OS was better in the p16+ group, similar to other previous studies.

Despite a low p16+ OPSCC in this study, the high density of CD8+ TIL was still associated with a favorable survival outcome and a lower rate of distant metastases. Additionally, there was a significant association between CD8+ TIL density and positive expression of p16. All OPSCCs with a higher CD8+ TIL density had a better OS than those with a lower CD8+ TIL, as well as in the p16− OPSCC subgroup. Previous studies found that the role of TIL in survival outcome and response to chemoradiation was demonstrated in HPV+ OPSCC, but there is still controversy about HPV- OPSCC [27,29,30]. Our results showing good OS in patients with high CD8+ TIL suggested that TIL could be a predictive prognostic factor, especially in the low-HPV-prevalence population. The role of TIL in cancer treatment has attracted research interests [31]. For example, the CheckRad-CD8 trial showed a promising result of induction chemoimmunotherapy and radioimmunotherapy (RIT) using a combination of durvalumab and tremelimumab in patients with an increase in CD8+ immune cells [32]. However, more studies are needed to assess the efficacy of immune checkpoint inhibitors based on the TIL.

Several immune checkpoints regulate T cell activity, including PD-1/PD-L1. Anti-PD-1, such as pembrolizumab and nivolumab, have been approved by the FDA to treat recurrent and metastatic HNSCC [9]. Tumor PD-L1 was associated with various prognoses in previous studies. For example, a meta-analysis by Yang et al. [33] found no significant differences in OS and progression-free survival (PFS) between PD-L1 positive and negative OPSCC; however, low CD8+ TIL HNSCC and worse OS was observed in the PD-L1 positive subgroup. However, another meta-analysis by Tang et al. [16] in the OPSCC subgroup did not show a significant correlation between PD-L1 expression and OS but better disease-free survival (DFS) in patients with higher expression of PD-L1. Our study showed 29.1% positive PD-L1 expression using a cut-off CPS ≥ 1 but found no statistical difference in OS between the positive and negative subgroups. However, there was a trend towards a better OS in the PD-L1 positive group.

The relationship of PD-L1 with TIL and HPV is also equivocal; some evidence demonstrated an association between PD-L1 and TIL or HPV [16,34,35]. Positive PD-L1 was associated with high CD8+ TIL in our study, but no association with p16 was observed. Despite the proven benefit of anti-PD-1 in those with recurrent and metastatic HNSCC [36], the prognostic value of PD-L1 IHC for survival was still controversial. Furthermore, since the rate of positive expression of PD-L1 in this study was remarkably low, further studies should be suggested to ratify the clinical outcomes of anti-PD-1 treatment in the Thai population.

Classification of TME into four types based on PD-L1 and TIL was suggested by Teng et al. [23] in 2015 and was applied to several cancer studies later. Our study showed the best survival outcome in type I TME (PD-L1+/TIL+) similar to the findings in melanoma, as well as in HNSCC, which showed the most favorable prognosis in type I (PD-L1+/TIL+) and type IV (PD-L1−/TIL+) [23,34,35,37]. The results suggested that type I and IV had better results according to TIL status, highlighting the importance of the immune response in tumor control.

We acknowledge some limitations in our study. The study is retrospective and has a short median follow-up time. The HPV-positivity status was not confirmed by PCR. However, the discordant rate of p16 and PCR for HPV DNA was less than 25% [7]. Moreover, due to the limitations of our medical database, we could not report other types of survival outcomes apart from OS.

Biomarkers that reflect tumor cell proliferation, such as p53 or Ki-67 (MIB-1), have been investigated in other studies with controversial results on survival and recurrent outcomes, although some reported Ki-67 as an unfavorable prognostic factor [38,39]. Some studies also showed an association between these biomarkers and cancer aggressiveness [40,41]. We did not examine the role of proliferative markers in this study. Nevertheless, it is another field to be explored.

Our results suggested a benefit of the integration of p16 and TIL in predicting survival outcomes and, possibly with further studies, treatment response. Therefore, we postulated that additional IHC staining of TILs would provide additional prognostic information on patient outcomes, especially in areas with low HPV and settings where HPV validation could not be performed. More studies are needed to confirm its value in the prediction of clinical response and to identify other potential prognostic biomarkers.

## 5. Conclusions

The prevalence of HPV-associated OPSCC in Thailand is low. Positive p16 and high CD8+ TIL density were associated with better OS in OPSCC patients, while PD-L1 status was not significantly related to OS. Of all types of TME, the adaptive immune resistance phenotype (type I, PD-L1+/TIL+) provided the best prognosis.

## Figures and Tables

**Table 1:** IHC results.

**CD8+ TIL, *n*(%)**	**(*n* = 159)**
<1%	59 (37.1)
≥1%, <5%	54 (34.0)
≥5%, <10%	19 (11.9)
≥10%	27 (17.0)
**PD-L1: CPS, *n*(%)**	**(*n* = 158)**
CPS < 1	112 (70.9)
CPS ≥ 1, < 20	31 (19.6)
CPS ≥ 20	15 (9.5)
**PD-L1: Intensity, *n*(%)**	**(*n* = 46)**
1+ (weak)	24 (52.2)
2+ (moderate)	19 (41.3)
3+ (strong)	3 (6.5)

**Table 2:** Association between p16, CD8+ TIL, and PD-L1.

	p16−	p16+	*p*-Value	PD-L1, CPS < 1	PD-L1, CPS ≥ 1	*p*-Value
CD8+ TIL, *n*(%)			0.005 *			0.039 *
<1%	51 (38.6)	8 (29.6)		48 (42.9)	11 (23.9)	
≥1%, <5%	50 (37.9)	4 (14.8)		39 (34.8)	15 (32.6)	
≥5%, <10%	14 (10.6)	5 (18.5)		11 (9.8)	8 (17.4)	
≥10%	17 (12.9)	10 (37.0)		14 (12.5)	12 (26.1)	
PD-L1, *n*(%)			0.596			
CPS < 1	94 (71.8)	18 (66.7)				
CPS ≥ 1	37 (28.2)	9 (33.3)				

* *p*-Value < 0.05.

**Table 3:** Relationships between patient characteristics and IHC.

	p16− (*n* = 133)	p16+ (*n* = 27)	*p*-Value	CD8+ TIL < 1% (*n* = 59)	CD8+ TIL ≥ 1% (*n* = 100)	*p*-Value	PD-L1; CPS < 1 (*n* = 112)	PD-L1; CPS ≥ 1 (*n* = 46)	*p*-Value
Age			0.045 *			0.754			0.445
Mean ± SD	61.12 ± 11.18	56.19 ± 13.42		60.58 ± 11.87	59.97 ± 11.61		59.73 ± 12.03	61.30 ± 10.94	
Sex, n (%)			0.066			0.325			0.156
Male	127 (95.5)	23 (85.2)		57 (96.6)	92 (92.0)		107 (95.5)	41 (89.1)	
Female	6 (4.5)	4 (14.8)		2 (3.4)	8 (8.0)		5 (4.5)	5 (10.9)	
Smoking status, n (%)			0.058			0.015 *			0.223
Yes	122 (93.1)	21 (80.8)		57 (98.3)	85 (86.7)		103 (92.8)	38 (86.4)	
No	9 (6.9)	5 (19.2)		1 (1.7)	13 (13.3)		8 (7.2)	6 (13.6)	
Alcohol use, n (%)			0.094			0.28			0.302
Yes	84 (66.7)	11 (44.0)		39 (70.9)	55 (57.9)		71 (67.0)	23 (53.5)	
Social drinking	16 (12.7)	6 (24.0)		6 (10.9)	16 (16.8)		14 (13.2)	8 (18.6)	
Never	26 (20.6)	8 (32.0)		10 (18.2)	24 (25.3)		21 (19.8)	12 (27.9)	
Subsite, n (%)			0.081			0.139			0.132
Base of tongue	60 (45.1)	11 (40.7)		27 (45.8)	43 (43.0)		44 (39.3)	26 (56.5)	
Tonsil	49 (36.8)	15 (55.6)		19 (32.2)	45 (45.0)		48 (42.9)	15 (32.6)	
Others	24 (18.0)	1 (3.7)		13 (22.0)	12 (12.0)		20 (17.9)	5 (10.9)	
Pathological grading, n (%)			0.698			0.815			0.069
Well diff.	18 (13.6)	3 (13.6)		9 (15.3)	12 (12.8)		16 (15.1)	5 (10.9)	
Moderately diff.	88 (66.7)	13 (59.1)		39 (66.1)	61 (64.9)		73 (68.9)	26 (56.5)	
Poorly diff.	26 (19.7)	6 (27.3)		11 (18.6)	21 (22.3)		17 (16.0)	15 (32.6)	
T stage, n (%)			0.144			0.317			0.89
T1	10 (7.5)	6 (22.2)		6 (10.2)	10 (10.0)		12 (10.7)	4 (8.7)	
T2	28 (21.1)	5 (18.5)		8 (13.6)	25 (25.0)		22 (19.6)	10 (21.7)	
T3	52 (39.1)	9 (33.3)		23 (39.0)	37 (37.0)		41 (36.6)	19 (41.3)	
T4	43 (32.3)	7 (25.9)		22 (37.3)	28 (28.0)		37 (33.0)	13 (28.3)	
N stage, n (%)			0.007 *			0.304			0.554
N0	27 (20.3)	4 (14.8)		16 (27.1)	15 (15.0)		23 (20.5)	8 (17.4)	
N1	19 (14.3)	11 (40.7)		11 (18.6)	19 (19.0)		24 (21.4)	6 (13.0)	
N2	62 (46.6)	6 (22.2)		22 (37.3)	45 (45.0)		44 (39.3)	22 (47.8)	
N3	25 (18.8)	6 (22.2)		10 (16.9)	21 (21.0)		21 (18.8)	10 (21.7)	
M stage, n (%)			0.213			0.040 *			0.035 *
M0	122 (91.7)	27 (100.0)		52 (88.1)	97 (97.0)		102 (91.1)	46 (100.0)	
M1	11 (8.3)	0 (0.0)		7 (11.9)	3 (3.0)		10 (8.9)	0 (0.0)	
Stage (AJCC 8th), n (%)			<0.001 *			0.954			0.578
I	3 (2.3)	6 (22.2)		4 (6.8)	5 (5.0)		8 (7.1)	1 (2.2)	
II	9 (6.8)	10 (37.0)		7 (11.9)	12 (12.0)		12 (10.7)	7 (15.2)	
III	24 (18.0)	11 (40.7)		12 (20.3)	23 (23.0)		25 (22.3)	10 (21.7)	
IV	97 (72.9)	0 (0.0)		36 (61.0)	60 (60.0)		67 (59.8)	28 (60.9)	
Stage (AJCC 7th), n (%)			0.368			0.461			0.62
I	3 (2.3)	2 (7.4)		3 (5.1)	2 (2.0)		5 (4.5)	0 (0.0)	
II	9 (6.8)	1 (3.7)		5 (8.5)	5 (5.0)		7 (6.3)	3 (6.5)	
III	24 (18.0)	3 (11.1)		8 (13.6)	19 (19.0)		20 (17.9)	7 (15.2)	
IV	97 (72.9)	21 (77.8)		43 (72.9)	74 (74.0)		80 (71.4)	36 (78.3)	

* *p*-Value < 0.05.

**Table 4:** Cox regression model for overall survival (OS).

	Univariate HR (95% CI)	*p*-Value	Adjusted HR (95% CI)	*p*-Value
Smoking status: yes (former/current)	3.46 (1.27–9.41)	0.015 *	2.48 (0.90–6.82)	0.079
Stage: III-IV	2.18 (1.01–4.70)	0.047 *	2.43 (1.11–5.32)	0.026 *
p16: negative	2.32 (1.24–4.33)	0.009 *	1.98 (1.05–3.71)	0.034 *
CD8+ TIL: low density	1.89 (1.28–2.78)	0.001 *	1.77 (1.18–2.67)	0.006 *
PD-L1: negative	1.33 (0.85–2.07)	0.211	1.13 (0.71–1.80)	0.603

* *p*-Value < 0.05.

## Data Availability

The data presented in this study are available on request from the corresponding author. The data are not publicly available due to privacy and ethical issues.
